# Congenital Erythropoietic Porphyria: A Rare Inherited Disorder

**DOI:** 10.7759/cureus.55558

**Published:** 2024-03-05

**Authors:** Porika Saikrishna, Gowrishankar Palaniswamy, Navya Pillikunte Doddareddy, Lyluma Ishfaq, Mah N Zargar, Fathima Wafa Eranhikkal, Sweta Sahu

**Affiliations:** 1 Dermatology, Guntur Medical College, Guntur, IND; 2 Medicine, Saveetha Medical College and Hospital, Chennai, IND; 3 Internal Medicine, Bangalore Medical College and Research Institute, Bengaluru, IND; 4 Medicine, Directorate of Health Services Kashmir, Srinagar, IND; 5 Medicine, Fatima Jinnah Medical University, Lahore, PAK; 6 Dermatology, Teaching University of Geomedi, Tablisi, GEO; 7 Surgery, Jagadguru Jayadeva Murugarajendra (JJM) Medical College, Davanagere, IND

**Keywords:** genetic variability, hematopoietic stem cell transplantation, erythrodontia, photosensitivity, porphyrinogens

## Abstract

Congenital erythropoietic porphyria (CEP), also known as Gunther's disease, is an uncommon autosomal recessive disorder caused by a mutation in the uroporphyrinogen III synthase gene. This mutation results in reduced enzyme levels in heme synthesis and the accumulation of pathogenic porphyrin isomers, uroporphyrin I and coproporphyrin I, leading to the clinical manifestations of CEP. Typically, CEP manifests shortly after birth with severe cutaneous photosensitivity, blistering, ulceration, and scarring. Erythrodontia, acro-osteolysis, and skeletal abnormalities are frequently present in conjunction with it. It can even manifest in utero as hydrops fetalis, with pink or red diaper staining as an early diagnostic clue. In this case, we present a 17-year-old male with complaints of discharge over the left foot, blisters upon sunlight exposure, extensive mottled pigmentation, excessive facial hair, mutilated fingers, and verrucous growth over the toes. Using a Wood's lamp revealed pink fluorescence of teeth and ulcers on the foot. Laboratory investigations demonstrated anemia, leukocytopenia, thrombocytopenia, and elevated urine uroporphyrin 1 and coproporphyrin 1 levels. Current treatment approaches include sun protection to avoid further skin damage, beta-carotene to reduce oxidative stress, and blood transfusions to manage anemia. Stem cell transplantation remains the sole curative therapy for this exceedingly rare condition. This case report underscores the rarity and complexity of CEP and emphasizes the challenges in its management.

## Introduction

Porphyrias represent a diverse group of inherited disorders stemming from abnormalities in heme synthesis. Each type of porphyria is linked to a specific enzyme defect within the heme biosynthesis pathway. These disorders are classified as hepatic or erythropoietic, depending on the primary site of porphyrin precursor and accumulation in the liver or the erythron (the part of the bone marrow that forms red blood cells). In erythropoietic porphyrias, increased levels of porphyrins are found in both the bone marrow and erythrocytes. Among the erythropoietic porphyrias, congenital erythropoietic porphyria (CEP) is notable for its distinct phenotype, differing significantly from others like erythropoietic protoporphyria (EPP) and X-linked protoporphyria (XLP). While EPP and XLP present similarly, CEP is characterized by more intense photosensitivity and debilitating effects [1].

CEP is a rare, autosomal recessively inherited disorder, affecting all ethnic groups. It results from a lack of uroporphyrinogen III synthase (UROS) enzyme activity, which is necessary for the fourth step of heme biosynthesis. The deficiency leads to abnormal porphyrinogens, namely uroporphyrinogen I and coproporphyrinogen I, in both immature and mature red blood cells in the bone marrow. These porphyrinogens spontaneously oxidize into corresponding porphyrins, which, when activated by light, cause damage to red blood cells and accumulate in various tissues and bones. The photoreactive nature of these porphyrins leads to severe skin damage upon exposure to sunlight and ultraviolet (UV) radiation, manifesting as blisters, vesicles, and increased skin fragility. While most cases of CEP are linked to mutations in the UROS gene, some instances have been associated with mutations in the GATA1 (globin transcription factor 1) gene, located on the X chromosome, further emphasizing this condition's genetic complexity and variability [2].

To date, approximately 250 cases of CEP have been documented in the scientific literature, exhibiting marked heterogeneity in clinical presentation. CEP is a rare disorder with a pan-ethnic distribution, displaying no predilection toward any specific demographic or racial group. It has been reported in individuals across a diverse array of ethnic backgrounds, including, but not limited to, Japanese, North Europeans, Hispanics, Indians, African Americans, and the Cree Indians. This widespread occurrence underscores the universal nature of the genetic anomaly responsible for CEP, transcending geographical, racial, and ethnic boundaries [2,3].

The clinical features of CEP vary with the age of onset. In utero, it may present as non-immune hydrops fetalis due to severe hemolytic anemia, or as fetal ascites. Postnatally, patients experience severe photosensitivity, while adults predominantly suffer from skin lesions. Dermatologically, CEP is characterized by intense sunlight sensitivity, leading to bullae and vesicles on exposed skin, prone to rupture and infection, resulting in scarring and deformities like finger loss. The accumulation of uroporphyrin I in red blood cells causes varying degrees of hemolysis in CEP, frequently resulting in secondary splenomegaly, anemia, leukopenia, and thrombocytopenia. Ocular manifestations exhibit blepharitis, ectropion, conjunctivitis, and loss of eyelashes and eyebrows, with risks of corneal scarring and potential blindness. Dental and skeletal manifestations in adults are severe, including osteolysis and mutilations. Bone changes in the calvarium, maxilla, mandible, and pelvic bones can be seen on X-rays. Finger contractures, acroosteolysis, and terminal phalanges atrophy can also be seen. Porphyrin deposits in the teeth and bones give them a unique color and fluorescence when exposed to UV light. Dental issues include incisor crowding short lips and inability to fully cover the teeth [3].

CEP poses several diagnostic challenges due to its rarity and varied presentation, making it difficult to recognize the condition based on clinical symptoms alone. Laboratory examinations, including biochemical testing for porphyrin levels in urine, feces, and blood, are required for differential diagnosis. Genetic tests identify mutations in the UROS gene, confirming the CEP diagnosis. However, the availability of genetic tests is not always straightforward, rendering this test inconclusive in some cases. The symptoms of photosensitivity and skin lesions may overlap with other dermatological conditions, necessitating a multidisciplinary approach involving dermatologists, hematologists, and geneticists [4].

CEP typically presents with severe photosensitivity, resulting in blistering, scarring, and mutilation of sun-exposed areas. Common clinical features include hemolytic anemia, splenomegaly, and hypertrichosis (excessive hair growth). Affected individuals may experience severe pain, bone deformities, and hepatosplenomegaly. Diagnosis often involves clinical presentation, biochemical testing, and genetic analysis. Elevated urinary and fecal levels of porphyrins and porphyrin precursors are typical findings. Molecular genetic testing can confirm mutations in the UROS gene [5]. Treatment aims to alleviate symptoms and prevent complications, emphasizing protective measures such as avoiding sunlight and using protective clothing and sunscreen. Blood transfusions may be necessary to manage anemia, and in severe cases, splenectomy may be considered. Hematopoietic stem cell transplantation (HSCT) has shown promise as a potential cure [6]. Ongoing studies explore gene therapy and enzyme replacement therapy as potential treatments and investigational therapies targeting heme biosynthesis and phototherapy are under exploration.

CEP significantly impacts the quality of life due to chronic pain, disfigurement, and social stigmatization associated with visible symptoms. Psychological support and counseling are crucial for affected individuals and their families. The prognosis varies depending on the symptoms' severity and supportive care availability. Early diagnosis and comprehensive management strategies can improve outcomes and reduce complications [5].

The objective of a CEP case report typically involves detailing the clinical presentation, diagnostic methods, treatment modalities, and outcomes of an individual with CEP. Case reports contribute valuable insights to medical knowledge by shedding light on rare diseases like CEP and elucidating their symptoms, complications, and management strategies. Furthermore, these reports aid healthcare professionals in recognizing and managing similar cases more effectively in the future.

## Case presentation

A 17-year-old adolescent male student from Guntur, Andhra Pradesh, India, presented with raw areas on both his feet and hands, accompanied by fluid-filled blisters that developed upon exposure to sunlight. He exhibited extensive scarring in both lower limbs and reported sensitivity to sunlight since his childhood. The patient was born into a non-consanguineous marriage with no family history of similar dermatological or photosensitive conditions. The patient's medical history did not include any chronic illnesses such as tuberculosis, leprosy, diabetes mellitus, hypertension, asthma, or thyroid abnormalities. Furthermore, there was no reported history of substance use, which could potentially confound the clinical picture.

On examination, notable findings included an ulcer with discharge on the patient's left foot, originating from a blister that had ruptured and subsequently ulcerated. A verrucous growth was also observed on the same foot. A systemic examination revealed pallor in the lower palpebral conjunctiva and pronounced mottled skin dyspigmentation (hyperpigmentation) on the face and extremities, largely attributed to UV light exposure. Milia was present around the nostril region. Additionally, corneal opacity suggesting corneal scarring, facial atrophy, and erythrodontia with normal hair texture were observed.

The distal limbs displayed hypertrophied skin scars and skin dyspigmentation as sequelae of repeated sun-exposure episodes. The photographs below depict severe mutilation and finger structure loss in the sun-exposed sections of the upper and lower extremities.

**Figure 1:**
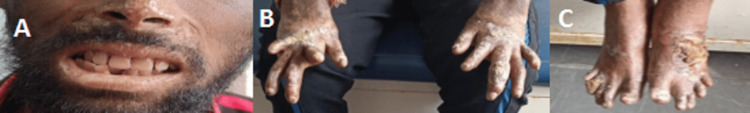
General appearance and facial skin changes, visible erythrodontia, and mutilated fingers A: visible erythrodontia, B: mutilated hand fingers, C: mutilated foot fingers

**Figure 2:**
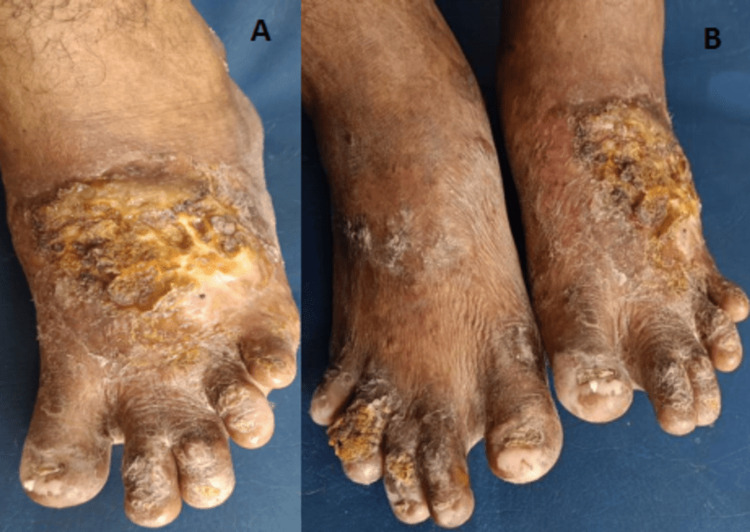
Hypertrophied scars in sun-exposed dorsal foot areas and verrucous growth over the toes A: verrucous growth over the toes, B: hypertrophied scars in sun-exposed dorsal foot areas

**Figure 3:**
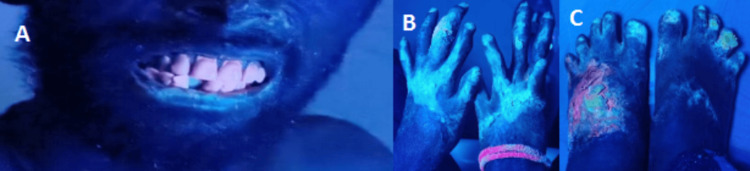
Wood's lamp examination findings, characteristic red-pink hue from teeth and blue hue from sun-exposed skin areas A: Wood's lamp examination of the face, B: Wood's lamp examination of the hands, C: Wood's lamp examination of the feet

These clinical manifestations, in conjunction with the patient's long-standing complaints related to sunlight exposure, posited a provisional diagnosis of CEP. A series of laboratory evaluations were performed to confirm the diagnosis. A routine blood panel revealed the presence of anemia, leukopenia, and thrombocytopenia. Urinalysis was notably significant for elevated concentrations of coproporphyrin I and uroporphyrin I. A Wood's lamp examination of the teeth and a hypertrophied scar on the dorsum of the left foot emitted a characteristic red-pink fluorescence, further supporting the diagnosis of CEP (Figures 1, 2, 3).

Treatment

The treatment plan focused on managing the condition and preventing further complications. The patient was advised to adhere to strict sun protection measures to mitigate additional skin damage. Emollients were prescribed to treat existing skin lesions, and antibiotics were recommended to prevent infections associated with the skin lesions. To ensure comprehensive care and monitor for potential complications associated with CEP, the patient was advised to undergo frequent follow-up assessments at the outpatient clinic.

## Discussion

CEP is an autosomal recessive genetic disorder affecting heme biosynthesis. It results in significantly reduced activity, rather than complete absence, of the cytosolic enzyme UROS, also known as URO-synthase. Also termed Günther's disease, the case was first described by Schultz in 1874 in a young male with a history of cutaneous sensitivity since birth [1]. CEP is inherited in an autosomal recessive manner or as an X-linked condition caused by mutations in the UROS or GATA1 genes [6]. The UROS gene produces the UROS enzyme, which has significantly reduced enzymatic activity in erythrocytes, ranging from undetectable to roughly 10% of the normal mean. This deficit causes the non-enzymatic conversion of hydroxymethylbilane to uroporphyrinogen I. As a result, uroporphyrinogen I is converted into coproporphyrinogen I, which accumulates because it cannot be further metabolized. These accumulating intermediates are oxidized to produce coproporphyrin I and uroporphyrin I, two harmful and non-physiological compounds associated with abnormalities involving various organs [5]. It has also been demonstrated that the activation of ALAS2 (aminolevulinate synthase 2), the initial and limiting enzyme in haem synthesis in erythroid cells, causes a more substantial production of porphyrins within the body [6].

Significant variation exists in the severity of the disease among unrelated patients, with milder adult cases characterized solely by cutaneous photosensitivity to nonimmune hydrops fetalis or lifelong transfusion dependence [3]. CEP typically presents at birth or during early infancy and is characterized by severe cutaneous photosensitivity leading to mutilating skin damage. Key dermatological symptoms include bullous lesions and heightened skin fragility, particularly in areas exposed to light, such as the hands, face, and feet. A notable initial symptom is the pink-to-dark red discoloration of urine in affected infants. The condition also manifests varying degrees of hemolysis, with severe cases necessitating regular blood transfusions. Patients often develop significant splenomegaly as a result of hemolytic anemia. Bone involvement is a consistent feature of CEP, marked by the thinning of bone structures and an increased risk of fractures.

Our case report features a 17-year-old man who has a history of blister production from sun exposure and scarring since childhood. On inspection, characteristic skin findings included an ulcer on the dorsum of the foot, skin thickening, hypo- and hyperpigmentation of the skin, hypertrichosis, and finger mutilation. Photosensitivity in CEP is primarily due to the circulation of porphyrins in the skin, which leads to free radicals upon exposure to light. These porphyrins have a distinct absorption peak in the 400 to 420 nm wavelength range, known as the Soret band, which lies close to the spectrum of long-wavelength UV light (UVA; range 315 to 400 nm). This sensitivity extends to sunlight and, to a lesser extent, artificial sources like fluorescent and incandescent indoor lighting. Notably, window glass, which blocks UVB but not UVA light, fails to offer protection against this effect. Light-activated porphyrins produce oxygen-free radicals, forming bullae and vesicles filled with serous fluid that fluoresces due to porphyrin content. These blisters are susceptible to rupture and infection. Recurring vesicle formation coupled with secondary infections can lead to persistent cutaneous scarring and deformities, potentially resulting in the loss of digits and facial features such as eyelids, nose, and ears. Additionally, the skin may exhibit thickening, variable pigmentation, and hypertrichosis, particularly on the face and extremities [7,8].

Hematological manifestations associated with CEP range from moderate to severe hemolytic anemia. The buildup of uroporphyrinogen I in the erythrocytes is most likely the cause of hemolysis. Common observations on the peripheral blood smear in severe cases of anemia include anisocytosis, poikilocytosis, polychromasia, basophilic stippling, and reticulocytosis. The laboratory results reveal the absence of haptoglobin, elevated levels of unconjugated bilirubin, and higher levels of fecal urobilinogen. Other findings include leukopenia and thrombocytopenia, which may be associated with bleeding diathesis. In addition, severe hemolytic anemia can result in secondary splenomegaly, whereas continuous transfusions might cause iron excess and other complications [5,8,9]. In our case, the comprehensive blood panel revealed the presence of anemia, leukopenia, and thrombocytopenia. Abdominal examination did not reveal any splenomegaly.

The eyes are particularly susceptible to photodamage due to porphyrin deposition in patients with CEP. This accumulation can result in corneal ulcers and subsequent scarring, potentially culminating in blindness. Other ocular complications include scleral necrosis, necrotizing scleritis, seborrheic blepharitis, keratoconjunctivitis, sclerokeratitis, and cicatricial ectropion. A notable diagnostic feature is the pink fluorescence of the perilimbal sclera under a Wood's light. Additionally, photomutilation can destroy eyelids, further exacerbating the risk of ocular damage [6,8]. Our case exhibited corneal opacity on ocular examination, which indicates long-term UV damage.

Porphyrin deposition in the dentition of patients with CEP results in a distinctive reddish-brown discoloration, known as erythrodontia, which exhibits fluorescence under a Wood's lamp. Furthermore, porphyrin accumulation in bone tissue leads to bone demineralization. This process is also associated with an expansion of bone marrow, a condition that can be observed as hyperplastic bone marrow in biopsy samples. Radiographic examinations typically reveal osteopenia and acro-osteolysis. Additionally, individuals with CEP who limit sun exposure to mitigate photodamage are at an increased risk of developing vitamin D deficiency. There is an increased risk of both spontaneous and traumatic fractures [6,8].

The diagnosis of CEP may be initially suspected in cases of non-immune hydrops fetalis, where the amniotic fluid surrounding the fetus exhibits a pink, dark-red, or brown hue. This fluid should be analyzed for porphyrin content. In neonates, pink to dark-red urine-stained diapers warrant immediate diagnostic investigations. The initial screening involves measuring total porphyrins in plasma or urine for porphyrias that manifest with blistering skin lesions. Elevated plasma or urine porphyrins necessitate comprehensive second-line testing to confirm the porphyria type or differentiate it from other conditions like liver disease, which can elevate urinary porphyrin levels. The biochemical diagnosis of CEP is confirmed by detecting markedly high levels of uroporphyrin I and coproporphyrin I in urine, erythrocytes, or amniotic fluid, along with increased fecal coproporphyrin I concentrations. Notably, CEP does not elevate urinary delta-aminolevulinic acid (ALA) and porphobilinogen (PBG) levels. A significant rise in total erythrocyte porphyrins, predominantly uroporphyrin I, is observed, and some patients show elevated zinc protoporphyrin.

The urine study of our case showed increased levels of coproporphyrin 1 and uroporphyrin 1, which confirmed the presence of CEP. Once CEP is suspected or identified through quantitative porphyrin analysis, molecular genetic testing for biallelic pathogenic variants in the UROS gene confirms the diagnosis. A hemizygous pathogenic variant in the X-linked GATA1 gene is identified in rare cases. Molecular genetic testing can encompass gene-targeted testing (single gene or multigene panel) or comprehensive genomic testing (exome sequencing, genome sequencing). Individuals with distinctive clinical features are likely to be diagnosed using gene-targeted testing, while those with less clear clinical presentations may require comprehensive genomic testing for diagnosis [5,8]. Due to financial constraints, genetic testing was not performed in our case, underscoring the reality that in areas with limited resources, the diagnosis of CEP relies heavily on clinical examination, thorough history-taking, and biochemical markers.

As of the current date, researchers have identified 18 distinct mutations in the UROS gene responsible for CEP. These genetic aberrations encompass a variety of types, including single-base substitutions, insertions and deletions, and splicing defects. Most of these mutations have been documented in only one or a few unrelated families. However, there are notable exceptions: the C73R, L4F, and T228M mutations represent a significant proportion of the mutant alleles studied, accounting for approximately 33%, 8%, and 7% of cases, respectively [3].

The p.C73R mutation in the UROS gene, representing the most prevalent mutation associated with CEP, demonstrates less than 1% enzyme activity compared to the wild-type allele. This substantial reduction in enzyme function is clinically significant, as individuals homozygous for the p.C73R mutation exhibit the most severe CEP phenotype, including non-immune hydrops fetalis and transfusion dependency from birth. In cases where patients are heteroallelic, possessing one allele with the p.C73R mutation and another with a mutation allowing minimal residual enzymatic activity (such as p.A69T), the resultant phenotype is severe or moderately severe. Conversely, heteroallelic patients with one mutation allowing for higher residual activities (e.g., mutations encoding p.V82F with 35% of normal activity, p.A104V with 7.7% of normal activity, and p.A66V with 14.5% of normal activity) tend to exhibit milder forms of CEP. This holds even if the accompanying allelic mutation is p.C73R or a mutation yielding no detectable activity, such as nonsense and frameshift mutations [6]. Furthermore, variants in the GATA1 gene, specifically the c.646C>T (p.Arg216Trp) pathogenic variant, also play a significant role in CEP pathology. This variant is believed to dramatically alter GATA 1 binding to UROS, leading to a considerable decrease in UROS activity, approximately 20% of the normal level. This finding underscores the complexity of the genetic landscape influencing the phenotypic variability in CEP [8].

Other diagnostic tests include fluorescence microscopy, which reveals red porphyrin fluorescence in intact erythrocytes and erythroid precursor cells, particularly visible in bone marrow smears under violet or blue light against a dark-field background. The distinct fluorescence of nuclei in erythrocyte precursor cells is specific for erythropoietic porphyria. A dermatopathologic examination reveals subepidermal blisters with superficial perivascular lymphocytic infiltrates, thickening, and hyalinization of blood vessels within the superficial vascular plexus, containing periodic acid-Schiff (PAS)-positive, diastase-resistant glycoproteins. Additionally, caterpillar bodies and eosinophilic linear structures in the roofs of bullae are observed. Direct immunofluorescence tests typically show linear C3 and immunoglobulin G staining around superficial vessels and the dermoepidermal junction [10].

The management of CEP necessitates a multidisciplinary team approach. Currently, the only curative treatment for CEP is bone marrow transplantation (BMT) or allogeneic hematopoietic stem cell transplantation, particularly for children with severe manifestations [11]. The primary management strategy for patients not undergoing transplantation involves rigorous avoidance of sun and light exposure, including UV and fluorescent light. Sunscreens containing zinc oxide or titanium oxide offer some protection, but they are not a substitute for strict light avoidance. Photodynamic therapy should not be administered to newborns with a confirmed diagnosis of CEP if they develop hyperbilirubinemia, to prevent severe cutaneous blistering. Protection of the eyes with wraparound sunglasses is recommended. Ocular complications such as corneal ulcers, scleritis, and blepharitis should be addressed with topical antibiotics. Corrective eyelid surgery alongside artificial tears and lubricants is essential for ectropion to prevent dry eyes and safeguard the cornea. Vitamin D supplementation is advised to counteract bone demineralization, and bisphosphonates may benefit individuals with osteoporosis. Skin trauma must be avoided; diligent wound care is essential to prevent infection in open blisters. Antiseptic measures, along with topical or oral antibiotics, are important to prevent complications like osteomyelitis and bone resorption, which can lead to mutilation. Laser hair removal is a viable option for managing facial hypertrichosis. Additionally, immunization against hepatitis A and B is recommended. This precautionary measure, however, often leads to a markedly restricted family and social life, significantly impacting the quality of life [4,8].

While beta-carotene has shown photoprotective effects in EPP, its efficacy in CEP is unproven [12]. Oral measures like activated charcoal and cholestyramine, which prevent the reabsorption of porphyrins, are often rendered impractical due to the large doses required. Oral alpha-tocopherol (vitamin E) and ascorbic acid (vitamin C) have been suggested to help reduce skin and blood cell damage by neutralizing reactive oxygen radicals [7]. Afamelanotide (Scenesse®), a synthetic melanocortin-stimulating hormone analog approved by the European Medicines Agency in 2014 for EPP patients, has not yet been investigated in CEP patients. This hormone, delivered subcutaneously, enhances melanin synthesis via the melanocortin-1 receptor. Studies in the United States and the European Union demonstrated that afamelanotide increased the duration of pain-free time in EPP patients and improved their quality of life. These findings, however, are yet to be replicated or explored in CEP patients [13].

In managing anemia in porphyria, a thorough evaluation is essential to identify and address deficiencies in iron, vitamin B12, or folate. For severe hemolysis, regular blood transfusions might be required. Administering chronic transfusions every two to four weeks, aiming for a hematocrit above 35%, can effectively suppress erythropoiesis and lower porphyrin production, thereby reducing porphyrin levels and photosensitivity. Parenteral or oral chelators are advised to counteract iron overload resulting from frequent transfusions [8].

Hydroxyurea has been observed to decrease erythropoiesis and bone marrow porphyrin production, improving photosensitivity, although it may not reduce the need for transfusions [14]. Hydroxychloroquine administration, plasmapheresis, and IV hematin infusion have not demonstrated significant efficacy in reducing porphyrin levels. Moreover, splenectomy may be a viable option for patients exhibiting splenomegaly, hemolytic anemia, thrombocytopenia, and leukopenia [8]. Iron deficiency, induced through the administration of iron chelators or via phlebotomies, has been shown to improve photosensitivity and hemolysis in a subset of affected individuals [15].

In a murine model of CEP, pharmacological chaperone therapy is being explored. This approach involves administering small-molecule drugs to enhance the residual activity of mutated enzymes with low activity or stability. Notably, the antimicrobial agent ciclopirox has been used as a chaperone to stabilize UROS, leading to a reversal of CEP-related manifestations such as abnormal uroporphyrin I levels in the blood, splenomegaly, and elevated liver porphyrins. However, this therapeutic strategy has not yet been applied to human subjects [16].

## Conclusions

This case report highlights the rare occurrence of CEP and outlines its diagnostic criteria and management strategies. CEP is characterized by various symptoms spanning dermatological, hematological, ocular, and skeletal abnormalities. The patient presented with a constellation of symptoms, including blisters caused by sun exposure since childhood, hypertrophic scars, hyperpigmentation in sun-exposed areas, corneal opacity, excessive facial hair, erythrodontia, and finger mutilation, all of which are characteristic of CEP. The detection of elevated urine uroporphyrin I and coproporphyrin I supported the diagnosis. Anemia, leukocytopenia, and thrombocytopenia were among the hematological abnormalities that accompanied the condition. The pink-red fluorescence of teeth under a Wood’s lamp examination further supported the diagnosis.

Therapeutic interventions were primarily aimed at mitigating symptom severity and included rigorous sun protection measures, the application of emollients to manage skin lesions, and antibiotic prophylaxis to prevent infections. The management strategy was complemented by regular outpatient follow-ups, enabling ongoing assessment and adjustment of the treatment plan as necessary. Early detection and management are critical for improving patient outcomes in this rare disease. This case emphasizes the importance of considering rare metabolic disorders in the differential diagnosis of patients presenting with photosensitivity and chronic skin lesions and the role of comprehensive clinical evaluation and targeted diagnostic testing in managing CEP. A multidisciplinary approach is necessary to manage the complications of CEP effectively. Stem cell transplantation has proven to be a successful treatment for this rare disorder. Further clinical trials are necessary to create standardized treatment guidelines for patients with CEP.
